# Purification of Alaskan Walleye Pollock (*Gadus chalcogrammus*) and New Zealand Hoki (*Macruronus novaezelandiae*) Liver Oil Using Short Path Distillation

**DOI:** 10.3390/nu6052059

**Published:** 2014-05-22

**Authors:** Alex C. M. Oliveira, Matthew R. Miller

**Affiliations:** 1 Alaska Sea Grant Marine Advisory Program, University of Alaska Fairbanks, 118 Trident Way, Kodiak, AK 99615, USA; E-Mail: acoliveira@alaska.edu; 2 The New Zealand Institute for Plant & Food Research Limited, P.O. Box 5114, Nelson 7010, New Zealand

**Keywords:** fish oils, molecular distillation, pollock, hoki, wiped film vacuum distillation

## Abstract

The beneficial health effects of a diet rich in *n*-3 long chain polyunsaturated fatty acids (*n*-3 LC-PUFA) have been extensively researched in recent years. Marine oils are an important dietary source of *n*-3 LC-PUFA, being especially rich in two of the most important fatty acids of this class, EPA (eicosapentaenoic acid; 20:5*n*-3) and DHA (docosahexaenoic acid; 22:6*n*-3). Oils rich in *n*-3 LC-PUFA are prone to oxidation that leads to loss of product quality. Alaskan pollock (Gadus chalcogrammus Pallas, 1814) and New Zealand’s hoki (*Macruronus novaezelandiae* Hector, 1871) are the highest volume fisheries of their respective countries. Both produce large quantities of fishery byproducts, in particular crude or unrefined *n*-3 LC-PUFA containing oils. Presently these oils are used as ingredients for animal feed, and only limited quantities are used as human nutritional products. The aim of this research was to investigate the applicability of short path distillation for the purification of pollock and hoki oil to produce purified human-grade fish oil to meet quality specifications. Pollock and hoki oils were subjected to short path distillation and a significant decrease in free fatty acids and lipid oxidation (peroxide and *para*-anisidine values) products was observed. Purified oils met the Global Organization for EPA and DHA Omega-3 (GOED) standard for edible fish oils.

## 1. Introduction

The beneficial health effects of *n*-3 long chain polyunsaturated fatty acids (*n*-3 LC-PUFA) are well established. There is evidence that *n*-3 LC-PUFA play a role in the treatment and possible prevention of cardiovascular diseases, hypertension, diabetes, arthritis, and other inflammatory and autoimmune diseases [1,2,3,4]. Marine oils are an important dietary source of *n*-3 LC PUFA, being especially rich in two of the most important fatty acids of this class, namely EPA (eicosapentaenoic acid; 20:5*n*-3) and DHA (docosahexaenoic acid; 22:6*n*-3). Due to its nutritional value, there is growing interest in refining fish oil from different marine sources for human consumption. Refined edible fish oil can be consumed in the form of a pharmaceutical (e.g., Omacor™, Lovaza™), or nutraceutical (e.g., fish oil capsules), or it can be added as an ingredient to boost levels of *n*-3 LC-PUFA in various food items such as baked goods, orange juice and yogurt [5].

Aquaculture is presently the major user of *n*-3 LC-PUFA oils. Therefore, the nutraceutical and pharmaceutical industries are in competition with aquaculture industries for access to *n*-3 LC-PUFA, and the increased demand for this marine oil has lead to an increase in the value of this commodity [6]. Currently, most of the fish oil used in aquaculture feeds is produced from non-food forage fish species from Peru and Northern Chile; Mexican and Central American Pacific coasts; the US Gulf and Atlantic coasts; Norway; Iceland; and other regions [7]. Most of these industrial fisheries are harvested at sustainable levels and increased oil production from these sources is unlikely [8]. A possible method for increasing the human intake of *n*-3 LC-PUFA is improving the quality and processing yields of oils rendered from abundant although presently underutilized marine byproduct streams.

Alaskan walleye pollock and New Zealand’s hoki are major world fisheries with large economic importance to their respective countries. Alaska walleye pollock, previous scientific name *Theragra chalcogramma* and recently reassigned to *Gadus chalcogrammus* [9], is the largest volume fishery in Alaska and estimated at over 1,000,000 tons per year [10]. The Bering Sea and Aleutian Islands, and the Gulf of Alaska pollock fisheries are considered sustainable and were certified by the Marine Stewardship Council (MSC) in 2005 and 2010. The combination of a high production volume and the year-round availability of pollock byproducts make these raw materials ideal for the production of human-grade fish oils [10]. Hoki (*Macruronus novaezelandiae*, family Merlucciidae) is the major commercial fishery in New Zealand. Hoki is a hake species and has MSC certification as being a well-managed species. The quota varies depending on stocking information and was set at 150,000 tons for the 2013–2014 fishing season. Hoki are found in the waters surrounding New Zealand, in particular the Chatham Rise, Cook Straight, around the Sub Antarctic islands as well as southeastern Australia. Both of these species store large amounts of oil in their liver making them a good source of *n*-3 LC-PUFA. Pollock liver oil has been reported to contain *n*-3 LC-PUFA concentrations of 23 g/100 g, with 5 g/100 g DHA and 15 g/100 g EPA [11]. Hoki liver oil contains similar concentrations of *n*-3 LC-PUFA (23 g/100 g), with a different ratio of DHA (12 g/100 g) to EPA (6 g/100 g) [12].

Hoki and pollock oils are rich in *n*-3 LC-PUFA and susceptible to lipid degradation processes which cause loss of the valuable LC-PUFA, and development of rancid odours and flavours [13]. Lipid oxidation is a degradative free radical reaction which can be triggered by many mechanisms such as singlet oxygen or peroxide radicals, from sources such as light and/or oxygen and catalysts such as iron [13]. Some important triggers of lipid oxidation in fish byproducts are iron from hemoglobin, as well as temperature and the presence of oxygen during processing [13]. Lipid oxidation products do not only impart unpleasant taste and smell to fish oils but they also exert cytotoxic and genotoxic effects [14,15]. Ingestion of these compounds may cause low density lipoprotein cytotoxicity [16], atherogenesis and atherosclerosis [17], and liver enlargement indicating nutrition-induced toxicity [18]. For these reasons, it is critical to monitor the quality and oxidative stability of edible fish oils. Oxidation products in crude marine oils, such as pollock and hoki liver oil, can be high, reducing the oil value and quality. Oxidation is measured by a series of tests. The Peroxide Value (PV) measures the primary products of lipid oxidation (lipid peroxides) while *para*-Anisidine Value (*p*-AV) is a method used to measure secondary products of oxidation (aldehydes and ketones). Acid Value (AV), often used as an indication of quality of the oil, quantifies free fatty acids (FFA) that have been cleaved from their parent molecules (e.g., triglycerides or phospholipids) as a result of hydrolytic breakdown. Cleavage of a FFA from a parent molecule, commonly known as lipid hydrolysis, results from increased enzymatic activity in the fish tissues post-mortem; the lipases triggering the degradation process are either innate to the organism or of bacterial origin. As there is no compulsory or regulatory qualitative parameters for lipid oxidation for marine oils to date, many marine oil processers use the Global Organization for EPA and DHA Omega-3 (GOED) voluntary monograph [19]. The 2012 GOED monograph states that for oils for human consumption, the following values are required: AV < 3 mg KOH/g; PV < 5 mEq/kg; *p*-AV < 20; and a resulting TOTOX of <26 (result of calculation, (2 × PV + *p*-AV) is also a requirement.) The GOED monograph also contains specifications for the maximum level of dioxins, PCBs and heavy metals with relevance to the process technology used in this study. In addition, the European Community has implemented hygienic, raw material quality and process requirements for fish oil intended for human consumption (Regulation (EC) 853/2004). The Codex Alimentarius Committee on Fats and Oils has recently started to assemble the Standard for Fish Oils and currently this document is at Step 2 [20].

In Alaska and New Zealand, large quantities of fishery byproducts are already utilized for the production of fishmeal and fish oil. However, most fish oil produced in Alaska and New Zealand is crude, only serving as an ingredient for animal/aquaculture feed. Food-grade fish oils can be produced from crude fish oils by including further processing steps that add value to marine byproducts for the respective fishing industries. Traditionally, fish oil purification is composed of four consecutive steps: degumming, neutralization, bleaching and deodorization. Degumming removes soluble and insoluble impurities such as proteins, phospholipids, waxes and trace metals [21]. Degumming is accomplished by washing the oil with an aqueous solution of an organic acid such as citric or phosphoric acid under mild heat [22]. Neutralization, often referred to as alkali refining, is used to remove FFA and this is accomplished by treating the degummed fish oil with sodium hydroxide (aqueous solution) under mild heat [21]. Bleaching the neutralized fish oil further purifies it by removing pigments, traces of soap, sulfur- and carbonyl-containing compounds, pigment breakdown products and trace metals [21]. Bleaching is accomplished by treating the oil with an adsorbent such as activated earth (bleaching clay), activated carbon, or chitosan [23]. Deodorization is the final purification step and consists of removing aldehydes and ketones that are responsible for the peculiar fish oil odor, which in most cases is not appealing to consumers. Aldehydes and ketones are formed during lipid oxidation, and this degradation of fatty acids may occur during raw material handling and storage, and/or during the rendering process. Since the late 1980’s, an additional step has been added in which the fish oil is also subjected to another step of molecular distillation to remove persistent organic pollutants (POP) [21]. In summary, the general objective of purification is to remove impurities that have negative health effects and detrimental sensorial and qualitative impacts on marine oils such as odor and taste.

Molecular distillation offers advantages for separation, purification and/or concentration of natural products, usually consisting of complex and thermally sensitive molecules such as fat-soluble vitamins and PUFA, because it minimizes losses caused by thermal degradation [24,25]. In this context, short-path distillation (SPD), provides an alternative to the traditional fish oil purification process by removing unwanted free fatty acids, deodorizing (removing aldehydes and ketones), and removing environmental contaminants under low pressure conditions [26,27,28]. One of the main advantages of using this technology, as compared with traditional fish oil purification steps, is that SPD does not require chemical treatments during processing, thus reducing processing effluents and decreasing the number of steps needed to refine fish oils. It is noteworthy to mention that it is expected that a majority of odorants, that is, low-molecular weight volatiles, in fish oils will be distilled off during short-path distillation due to the low-pressure used in the SPD system. However, SPD has its limitations and will not remove most pigments or heavy metals and therefore, cannot replace all physical refining steps used in traditional fish oil refining when either depigmentation or removal of heavy metals are required. The aim of this work was to investigate the applicability of short-path distillation to refine crude pollock and hoki oils to produce purified human-grade fish oil that meet GOED quality specifications.

## 2. Experimental Section

### 2.1. Materials

Pollock oil was produced in November of 2008 onboard the *F/T American Triumph* (American Seafoods Group) during the Bering Sea Pollock season. The pollock oil, produced at sea, was rendered from a mixture of fresh byproducts using a sequence of three inline horizontal contherm heat exchangers operated at 85–90 °C, and product cook time was less than 2 min. The cooked material was then separated into oil, water, and a protein sludge using a three-phase centrifuge operated at about 85 °C. Ascorbyl palmitate was used as an antioxidant. Ascorbyl palmitate (Sigma Aldrich, St. Louis, MI, USA) was mixed into the centrifuged crude Pollock oil, immediately after rendering, at a ratio of 250 mg/kg [29]. The oil was stored in 25 kg containers fitted with screw-top caps and frozen at −20 °C. Pollock oil was received frozen at the Kodiak Seafood and Marine Science Center (Kodiak, Alaska) and kept at −30 °C until used.

Hoki oil was produced by Sealord Group Ltd. (Nelson, New Zealand) at their rendering plant on 6 August 2006. Barox™ (Kemin, Des Moines, IA, USA) was used as an antioxidant and was added at 750 ppm immediately after rendering. The oil was kept at −40 °C until shipment to Kodiak, Alaska, in March of 2011.

### 2.2. Purification of Fish Oil Using Short-Path Distillation

**Figure 1:**
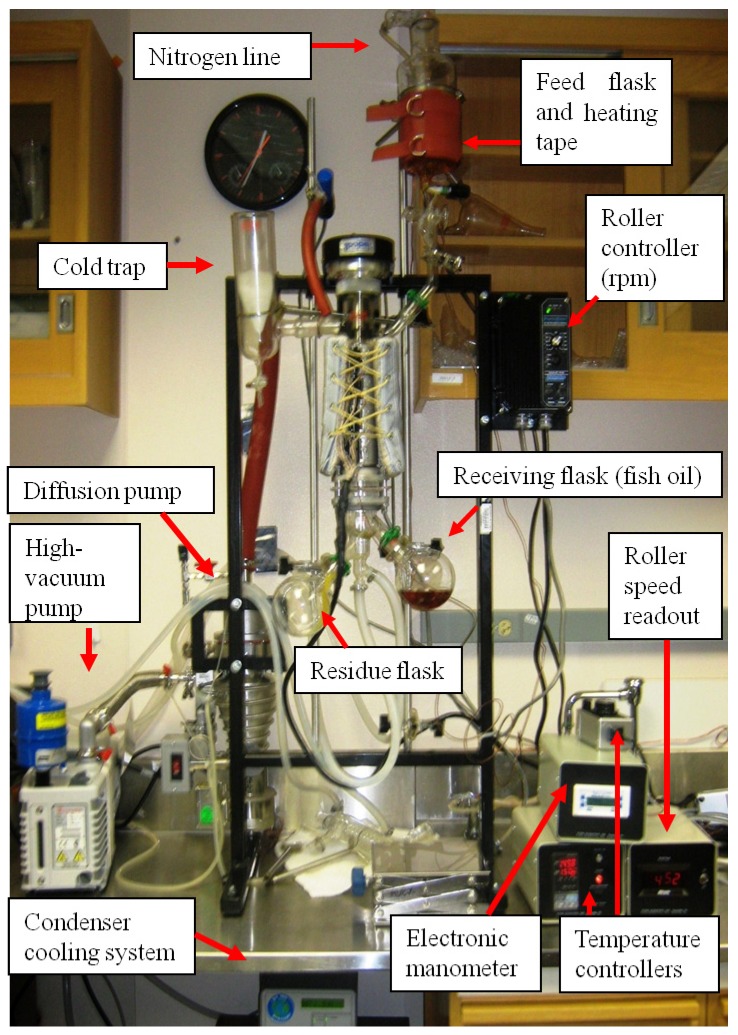
Short-path distillation (SPD) system.

The SPD process was conducted using a combination of processing variables in a sequence similar to previously reported [30,31,32]. The SPD apparatus (Figure 1) consisted of a Pope 2ʺ Wiped-film Still (Pope Scientific Inc., Saukville, WI, USA) connected to a Diffstak^®^ Mk2 diffusion pump model 63/150 (BOC Edwards, Crawley, West Sussex, UK) and also to a high-vacuum pump model RV3 (BOC Edwards). A Penta-Drive DC Meter Speed Control (Pope Scientific Inc.) for controlled rotation of the carbon blades in the evaporator was set at 450 or 500 revolutions per minute and digitally displayed by a RPM meter (Minarik, Aneheim, CA, USA). The surface area of the wiped film is 0.033 m^2^, the evaporator was contained in a heated jacket (Pope Scientific Inc., Saukville, WI, USA), and temperature of the evaporator was digitally controlled with a Digital Indicating Controller model UT35A (Yokogawa Electronic Corporation, Sugarland, TX, USA) and system pressure was monitored by a Digital Pressure Monitor (Kurt J. Lesker Company, Philadelphia, PA, USA). Distillation was conducted in a two-step procedure. The SPD cold-trap was cooled with dry-ice in acetone.

Pollock oils were purified using the SPD system during the summer of 2009 and hoki oils were purified in the spring of 2012. A portion of 1500 mL of either crude fish oil was added to the graduated feed flask and the heat tape enclosing the flask was set to 60 °C. The first distillation (first degassing pass) parameters were as follows: internal condenser temperature 55 °C; evaporator temperature 150 °C; feeding rate 360–480 mL/h; roller speed 450 rpm (hoki) or 500 rpm (pollock) and vacuum 0.05–0.06 mbar. The degassed oil was used immediately or stored in a sealed vessel under nitrogen at 5 °C for a maximum of 24 h. The main fish oil distillation (second pass) parameters were as follows: condenser temperature 55 °C; evaporator temperatures were 190 °C, 200 °C or 210 °C; feeding rate 360–480 mL/h; roller speed 450 rpm (hoki) or 500 rpm (pollock) and vacuum 0.01–0.02 mbar. The refining yields were determined gravimetrically, with about 300 g of oil trialed for each temperature parameter. The purification process was repeated three times for each type of oil and yielded nine purified oil samples (three independent oil replicates for each evaporator temperature tested).

### 2.3. Proximate and Oxidation Analysis

Rancidity and oxidation of fish oils were assessed in accordance to AOCS official methods including Acid Value (AOCS Official Method Cd 3d-63, [33]), Peroxide Value (AOCS Official Method Cd 8-53, [34]) and *para*-Anisidine Value (AOCS Official Method Cd 18-90, [35]). Reported TOTOX values were calculated via the equation TOTOX = (2 × PV) + *p*-AV [13]. Water content was measured by the Karl-Fisher method using an automated tritrator; values were generally <0.02%.

### 2.4. FAME Analysis

Fatty acid methyl esters (FAME) were prepared using KOH and methanol [36]. FAME were transferred into 1.5 mL snap-cap amber GC vials (Agilent Technologies, Wilmington, DE, USA) and immediately analyzed. An internal standard, tricosanoic methyl ester (23:0), was used for quantification. Fatty acid profiles were determined with a GC model 6850 coupled to a flame ionization detector (Agilent Technologies, Wilmington, DE, USA) fitted with a DB-23 (60 m × 0.25 mm id., 0.25 μm film) capillary column (Agilent Technologies, Wilmington, DE, USA). An autosampler performed the GC injections and injection volume was 1 μL. The chromatographic conditions were as previously described [37]. Unsaponifiable matter was calculated from the initial recorded weight of the oil (~20 mg) used for methylation, compared to the total lipid converted to methyl esters (mg FAMES). FAME were quantified in mg/g oil by using an internal standard and also a five-point calibration curve for all fatty acids included in the Supleco 37 mix (Sigma Aldrich, St. Louis, MI, USA) [38].

### 2.5. Statistical Analysis

Mean values were reported plus or minus standard error of the mean. In cases where data was reported in percentage composition, values were transformed using arcsin function to yield normalized data prior to statistical analysis. Normality and homogeneity of variance were confirmed and a comparison between means was achieved by one-way analysis of variance (ANOVA). Multiple comparisons were achieved by Tukey-Kramer HSD (honestly significant difference). Significance was accepted as probabilities of 0.05 or less. Statistical analysis was performed using SPSS^®^ statistics 17.0 and GenStat version 14 software.

## 3. Results

### 3.1. Peroxide Value (PV)

**Figure 2:**
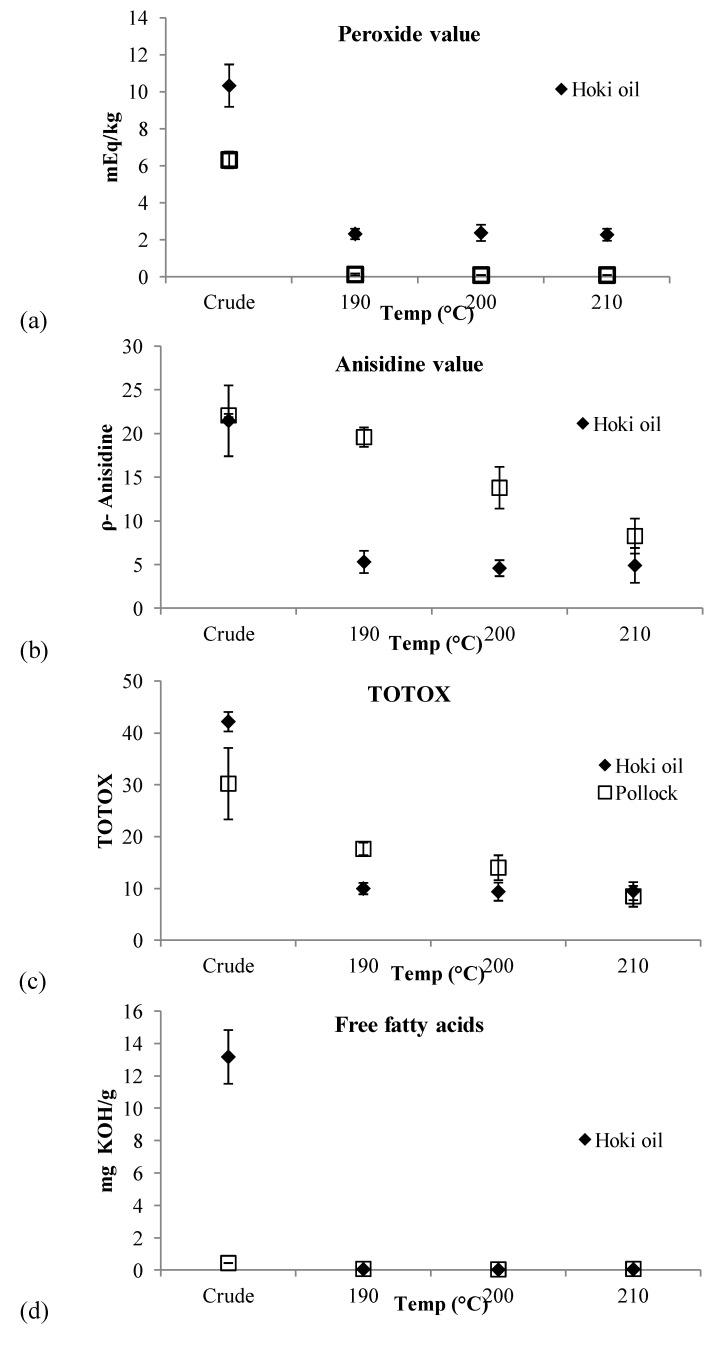
Quality indices of crude pollock (*n* = 3) and hoki (*n* = 3) oils, purified by short path distillation with variable evaporator temperatures (190 °C, 200 °C and 210 °C). (**a**) Peroxide Values; (**b**) *para*-Anisidine Values; (**c**) TOTOX Values; and (**d**) free fatty acids (FFA) Values.

Figure 2a depicts the PV of crude hoki and pollock oils together with values determined after oils were subjected to molecular distillation with set evaporator temperatures of 190, 200 or 210 °C. The crude pollock oil, with an initial PV of 6.32 ± 0.45 mEq/kg, was purified by molecular distillation with set evaporator temperatures of 190 °C (PV of 0.13 ± 0.06 mEq/kg), 200 °C (PV of 0.10 ± 0.00 mEq/kg) and 210 °C (PV of 0.10 ± 0.00 mEq/kg) (Figure 2a). PV of the three refined pollock oils did not statistically differ from each other and were all significantly (*p* < 0.001, *f* = 561.1) lower than the crude pollock oil. The crude hoki oils had significantly (*p* < 0.001, *f* = 113.6) higher PV, 10.33 ± 1.15 mEq/kg, than the values measured for any of the distilled hoki oils (190 °C 2.32 ± 0.28 mEq/kg; 200 °C 2.38 ± 0.44 mEq/kg; 210 °C 2.28 ± 0.33 mEq/kg). Significant differences were not observed in the PV of hoki oils distilled at the different evaporator temperatures tested.

### 3.2. Para-Anisidine Value (p-AV)

Figure 2b depicts the *p*-AV of crude hoki and pollock oils together with values determined after oils were subjected to molecular distillation with set evaporator temperatures of 190, 200 or 210 °C. The *p*-AV of crude pollock oils (20.09 ± 0.19) were significantly higher (*p* < 0.001, *f* = 41.7) than values determined for the higher temperature distilled oils (200 °C 13.81 ± 2.39 and 210 °C 8.28 ± 2.01). Crude hoki oils had significantly (*p* < 0.001, *f* = 37.8) higher *p*-AV (21.48 ± 4.06) than the values measured for hoki oils distilled using all evaporator temperatures: 190 °C (5.33 ± 1.27), 200 °C (4.61 ± 0.92) or 210 °C (4.93 ± 2.00). The *p*-AV of hoki oils subjected to molecular distillation at the tested evaporator temperatures were not significantly different.

### 3.3. TOTOX Value

Figure 2c depicts the TOTOX values of crude hoki and pollock oils together with values determined after oils were subjected to molecular distillation with set evaporator temperatures of 190, 200 or 210 °C. The TOTOX values of crude pollock oils (30.22 ± 6.90) were significantly (*p* < 0.001, *f* = 17.63) higher than those of their respective distilled counterparts at any of the tested SPD evaporator temperatures. A significant reduction in the TOTOX values (*p* < 0.001, *f* = 17.63) of pollock oils distilled at 210 °C (8.48 ± 1.22) was observed when these were compared to the TOTOX values of pollock oils distilled at either 190 °C (19.87 ± 2.39) or 200 °C (14.01 ± 2.00); however, the TOTOX values of oils distilled at the two lower temperatures did not significantly differ from one another. Crude hoki oils had significantly (*p* < 0.001, *f* = 296.9) higher TOTOX values (42.15 ± 1.87), than those of their respective distilled counterparts at any of the tested SPD evaporator temperatures. Regardless of the evaporator temperature tested, the TOTOX values of distilled hoki oils were not significantly different (190 °C 9.98 ± 1.10; 200 °C 9.37 ± 1.74; 210 °C 9.49 ± 1.72).

### 3.4. Acid Value (AV)

Figure 2d depicts the AV of crude hoki and pollock oils together with values determined after oils were subjected to molecular distillation with set evaporator temperatures of 190, 200 or 210 °C. The AV values of crude pollock oils (0.45 ± 0.01 mg KOH/g) were significantly higher (*p* < 0.001, *f* = 1634.6) than values determined for distilled pollock oils, regardless of tested SPD evaporator temperatures: 190 °C (0.09 ± 0.01 mg KOH/g), 200 °C (0.06 ± 0.01 mg KOH/g) or 210 °C (0.09 ± 0.01 mg KOH/g). The AV of crude hoki oils averaged 13.18 ± 1.65 mg KOH/g of oil and were significantly (*p* < 0.001, *f* = 187.4) higher than the values obtained for distilled hoki oils (190 °C 0.06 ± 0.00 mg KOH/g, 200 °C; 0.04 ± 0.03mg KOH/g; 210 °C 0.06 ± 0.00 mg KOH/g). A significant difference was not observed in the AV values of distilled hoki oils.

### 3.5. Fatty Acid Profiles

The FA profiles of the crude pollock oil and three distilled fractions are shown in Table 1. In general the amount of fatty acids in the distilled fractions was significantly higher due to the reduction in unsaponifiable matter, in particular in the 210 °C fraction. The crude pollock oil had significantly higher (*p* < 0.001, *f* = 45.63) unsaponfiable matter (24.01 ± 0.94) than the oils from the three different treatments (190 °C 15.95 ± 1.76; 200 °C 17.16 ± 0.27; 210 °C 10.54 ± 0.27). The two most abundant (>10.0 g/100 g) fatty acids observed in the pollock crude oils were long chain monounsaturated fatty acids (LC-MUFA): (20:1*n*-11, 12.2 g/100 g and 22:1*n*-11, 12.0 g/100 g). The n-3 LC PUFA were also present: DHA (4.0 g/100 g) and EPA (7.8 g/100 g). The most abundant FA class was MUFA (46.5 g/100 g) with the remainder being composed of saturated (SFA 13.8 g/100 g) and PUFA (15.6 g/100 g). The *n*-3 PUFA made up a majority (91%) of the PUFA fraction.

**Table 1:** Fatty acid (g/100 g oil) and unsaponifiable matter content of crude pollock oils (±SD) and pollock oils (±SD) purified by short-path distillation (SPD) with variable evaporator temperatures (190 °C, 200 °C, 210 °C).

Fatty acids ^1^ (g/100 g)	Crude oil (±SEM)	SPD 190 °C (±SEM)	SPD 200 °C (±SEM)	SPD 210 °C (±SEM)	
14:0	3.85 ± 0.09a	4.00 ± 0.13a,b	3.96 ± 0.06a,b	4.35 ± 0.02b	
16:0	6.61 ± 0.12a	7.10 ± 0.20a	7.00 ± 0.07a	7.66 ± 0.01b	
16:1*n*-7	7.70 ± 0.14a	8.25 ± 0.23a,b	8.13 ± 0.08a	8.88 ± 0.00b	
18:1*n*-9t	1.59 ± 0.02a	1.73 ± 0.04b	1.71 ± 0.01b	1.85 ± 0.00c	
18:1*n*-9	4.15 ± 0.06a	4.55 ± 0.11b	4.47 ± 0.02b	4.84 ± 0.01c	
18:1*n*-7	2.50 ± 0.03a	2.75 ± 0.06b	2.70 ± 0.01b	2.93 ± 0.00c	
20:1*n*-11	12.17 ± 0.14a	13.65 ± 0.25b	13.45 ± 0.02b	14.49 ± 0.04c	
20:1*n*-9	3.45 ± 0.03a	3.83 ± 0.08b	3.80 ± 0.02b	4.04 ± 0.00c	
22:1*n*-11	12.01 ± 0.07a	13.67 ± 0.19b	13.48 ± 0.05b	14.46 ± 0.14c	
18:3*n*-3	0.25 ± 0.01a	0.28 ± 0.01a,b	0.27 ± 0.00a,b	0.29 ± 0.00b	
18:4*n*-3	1.22 ± 0.02a	1.34 ± 0.03b	1.32 ± 0.01b	1.42 ± 0.00c	
20:4*n*-3	0.29 ± 0.00a	0.34 ± 0.01b	0.33 ± 0.00b	0.36 ± 0.00c	
20:5*n*-3	7.83 ± 0.07a	8.77 ± 0.17b	8.62 ± 0.01b	9.26 ± 0.01c	
22:2*n*-6	0.42 ± 0.01a	0.45 ± 0.01a,b	0.46 ± 0.00a,b	0.48 ± 0.00c	
22:5*n*-3	0.63 ± 0.00a	0.71 ± 0.01b	0.70 ± 0.00b	0.75 ± 0.01c	
22:6*n*-3	4.00 ± 0.02a	4.55 ± 0.07b	4.48 ± 0.01b	4.79 ± 0.04c	
Other SFA	3.42 ± 0.09a	3.76 ± 0.11a,b	3.70 ± 0.05a,b	4.02 ± 0.00c	
Sum SFA	13.88 ± 0.29a	14.85 ± 0.44a,b	14.66 ± 0.18a	16.02 ± 0.01b	
Other MUFA	2.94 ± 0.03a	3.28 ± 0.06b	3.24 ± 0.01b	3.49 ± 0.02c	
Sum MUFA	46.50 ± 0.50a	51.71 ± 0.99b	50.97 ± 0.09b	54.97 ± 0.20c	
Sum PUFA	15.61 ± 0.15a	17.49 ± 0.35b	17.21 ± 0.02b	18.47 ± 0.06c	
Sum *n*-3	14.22 ± 0.12a	15.98 ± 0.31b	15.71 ± 0.02b	16.87 ± 0.06c	
Sum Unsaponifiable matter	24.01 ± 0.94a	15.95 ± 1.76b	17.16 ± 0.27b	10.54 ± 0.27c	

Note: ^1^ Values are means ± SEM, *n*-3. Means in a row with different letters differ significantly as determined by Tukey-Kramer HSD, *p* < 0.01. SFA, Saturated fatty acids; MUFA, Monounsaturated fatty acids; PUFA, Polyunsaturated fatty acids.

In hoki oil, there were only minor differences observed between FA profiles of crude and distilled hoki oils (Table 2). The three most abundant (>10.0 g/100 g) fatty acids observed in the hoki oils were oleic acid (OA, 18:1*n*-9, 23.0 g/100 g), palmitic acid (PA, 16:0, 17.0 g/100 g) and DHA (22:6*n*-3, 12.0 g/100 g). Other important fatty acids were 20:1*n*-9 (7.0 g/100 g) and EPA (20:5*n*-3, 6.5 g/100 g). The most abundant FA class was MUFA (44.0 g/100 g) with the remainder being composed of SFA (24.8 g/100 g) and (PUFA 24.0 g/100 g). The *n*-3 PUFA made up a majority (85%) of the PUFA fraction. There was no statistical difference in the unsaponifiable matter in the crude and purified hoki oils.

**Table 2:** Fatty acid (g/100 g oil) and unsaponifiable matter content of crude hoki oils (±SD) and hoki oils (±SD) purified by short-path distillation (SPD) with variable evaporator temperatures (190 °C, 200 °C, 210 °C).

Fatty Acid ^1^ (g/100 g)	Crude oil (±SEM)	SPD 190 °C (±SEM)	SPD 200 °C (±SEM)	SPD 210 °C (±SEM)	
14:0	3.56 ± 0.01a	3.52 ± 0.10a	3.51 ± 0.05a	3.54 ± 0.07a	
16:0	17.04 ± 0.08a	16.84 ± 0.49a	16.92 ± 0.29a	17.04 ± 0.51a	
18:0	3.20 ± 0.03a	3.16 ± 0.06a	3.19 ± 0.06a	3.21 ± 0.09a	
16:1*n*-7	4.76 ± 0.02b	4.74 ± 0.06a,b	4.72 ± 0.04a	4.84 ± 0.02a,b	
18:1*n*-9t	0.47 ± 0.01a	0.48 ± 0.02a	0.48 ± 0.01a	0.49 ± 0.01	
18:1*n*-9c	22.91 ± 0.17a,b	22.81 ± 0.29a	22.82 ± 0.03a	23.32 ± 0.08b	
18:1*n*-7	3.14 ± 0.02a	3.07 ± 0.09a	3.11 ± 0.06a	3.21 ± 0.01a	
20:1*n*-9	7.03 ± 0.07a	6.98 ± 0.08a	7.00 ± 0.04a	7.12 ± 0.07a	
20:1*n*-7	0.32 ± 0.00a	0.31 ± 0.01a	0.31 ± 0.01a	0.33 ± 0.00a	
22:1*n*-11	3.20 ± 0.05a	3.19 ± 0.04a	3.18 ± 0.02a	3.23 ± 0.06a	
24:1*n*-9	1.14 ± 0.01a	1.13 ± 0.02a	1.13 ± 0.02a	1.13 ± 0.02a	
18:2*n*-6	2.61 ± 0.01a	2.62 ± 0.04a	2.61 ± 0.02a	2.68 ± 0.02a	
18:3*n*-3	0.79 ± 0.01a	0.78 ± 0.01a	0.76 ± 0.01a	0.77 ± 0.01a	
18:4*n*-3	0.93 ± 0.02a	0.94 ± 0.01a	0.93 ± 0.01a	0.96 ± 0.02a	
20:5*n*-3	6.43 ± 0.06a	6.48 ± 0.13a	6.46 ± 0.08a	6.68 ± 0.15a	
22:5*n*-6	2.04 ± 0.02a	2.10 ± 0.17a	2.03 ± 0.05a	2.06 ± 0.06a	
22:6*n*-3	11.88 ± 0.12a	11.99 ± 0.25a	11.94 ± 0.15a	12.32 ± 0.36a	
Total SFA	24.79 ± 0.06a	24.87 ± 0.07a	24.68 ± 0.12a	24.81 ± 0.17a	
Total MUFA	43.97 ± 0.02a	43.76 ± 0.05a	43.76 ± 0.02a	44.69 ± 0.07a	
Total *n*-3 PUFA	20.04 ± 0.06a	20.28 ± 0.20a	20.09 ± 0.04a	20.79 ± 0.11a	
Total PUFA	23.50 ± 0.03a	23.77 ± 0.04a	23.66 ± 0.09a	24.46 ± 0.12a	
Sum Unsaponifiable matter	7.74 ± 0.24a	7.60 ± 0.29a	7.90 ± 0.29a	6.04±0.97a	

Note: **^1^** Values are means ± SEM, *n*-3. Means in a row with different letters differ significantly as determined by Tukey–Kramer HSD, *p* <0.01. SFA, Saturated fatty acids; MUFA, Monounsaturated fatty acids; PUFA, Polyunsaturated fatty acids.

## 4. Discussion

### 4.1. Removal of Lipid Oxidation Products by SPD

The SPD system effectively removed the markers for lipid oxidation in both pollock and hoki oils. The primary products of oxidation, measured as PV, were significantly reduced in the distilled oils from both fish species examined and met the GOED monograph of quality marine oil. The final PV results in distilled pollock oils were lower than in distilled hoki oil, regardless of distillation temperature. Neither oil type showed a graded reduction in the PV with increasing temperature of the evaporator in the SPD system. Crude hoki oil had higher (10.3 ± 1.2 mEq/kg) starting PV values than those recorded for crude pollock oil (6.3 ± 0.5 mEq/kg). The higher PV level may reflect the differences in storage time (hoki oil 5.5 years, pollock oil 10 months) or differences in crude oil processing conditions. Previous work on better quality commercial oils showed PV values reduced from 1.8 mEq/kg to 0.7 mEq/kg [26] and all distilled oils studied had values in this range, moreover pollock and hoki oils had much lower PV than the maximum recommended in the GOED voluntary monograph (<5 mEq/kg) [19]. There was an 80% reduction in lipid peroxides between crude and distilled hoki oils (2.38–2.36 mEq/kg), while this reduction was 97% for the pollock oil (<0.14 mEq/kg). Further work is needed to understand the efficiency of the SPD system in removing peroxides from fish oils, however, our findings indicate the initial content of peroxides in the crude oil appeared to have a significant impact in the values found in distilled oils. Further adjustments to distillation process variables need to be investigated, which can counterbalance variability in the initial levels of peroxides in crude fish oil, to yield consistent rate of reduction of peroxides from crude to distilled oils. For instance, a slower flow of the oil through the evaporator may yield lower peroxide values in the distilled product because peroxides will decompose into secondary oxidation products that may be distilled off depending on molecular weight. On the other hand, prolonged exposure to high temperatures even under extremely low pressures may promote removal of other constituents of the oil that have antioxidant properties, such as fat soluble vitamins, which may be desirable in the final product.

A previous study on marine oil purification on a larger SPD (0.06 m^2^, double the size of our wiped surface area) showed reductions in PV, *p*-AV and TOTOX over a series of experiments that was greater than reductions observed for traditional activated carbon treatments [26]; this study demonstrated that reduction in PV is related to flow rate and evaporator temperature [26]. A commercial scale SPD (3m^2^ wiped surface area, 350 Lh^−1^ flow) study showed PV reduction, but did not show as pronounced reduction in oxidation parameters (14%–32% reduction in PV and 0%–21% reduction in *p*-AV) as we have demonstrated (76%–98% reduction in PV and 12%–78% reduction in *p*-AV) [28]. Our slow flow rate (0.36–0.48 Lh^−1^), high evaporator temperatures (190–210 °C), and small surface area (0.033 m^2^) may have resulted in greater reductions in PV. It is difficult to make comparisons between studies using different SPD; however, differences based on flow rate per m^2^ of evaporator surface area will give good indication of residence time of oil. In our study we had an estimated residence time of 10.9–14.5 Lh^−1^·m^−2^, while the larger scale study was ten-fold greater at 116.7 Lh^−1^·m^−2^ [28]. Traditional fish oil refining also includes a bleaching step to remove colored compounds and oxidation products. If reduction in oil color is desirable, then fish oils should be subjected to beaching prior to SPD and this likely further reduces the PV and *p*-AV values of finished product.

The SPD was an effective process to also remove secondary lipid oxidation products (carbonyl-containing compounds) that were measured using the *p*-AV method for both oils studied. In the pollock oils the results demonstrated an evaporator temperature effect, with pollock oils distilled at 210 °C showing the greatest reduction in *p*-AV when compared with values for oils distilled at either 190 or 200 °C. All distilled oils had *p*-AV below the GOED voluntary monograph recommended level of <20; however, pollock oil distilled at 190 °C had an average *p*-AV (19.6 ± 1.1) that was very close to this limit. These results suggest that within the temperature range studied, an evaporator temperature of 210 °C is preferred for distilling crude pollock oils that contained ascorbyl palmitate when using the SPD system; while for crude hoki oils any of the tested temperatures would be suitable for removing secondary products of oxidation. One potential confounding variable is ascorbyl palmitate that was added to crude pollock oil (250 ppm) for its established antioxidant properties [27], which was absent in the crude hoki oil. The hoki oil had Barox™ added post rendering as an antioxidant at a maximum of 750 ppm. A weakness in our study with this regard is the lack of data regarding quantity of ascorbyl palmitate or Barox removed from pollock and hoki oils as a result of distillation at the three tested evaporator temperatures. Further investigations are necessary to determine the influence of these additives in fish oils as it pertains to distillation efficiency of secondary products of oxidation using the SPD system.

The TOTOX values for distilled hoki and pollock oils, regardless of the evaporator temperatures tested, fell well under that required by the GOED monograph (TOTOX < 26). As TOTOX values are a function of PV and *p*-AV, not surprisingly the results for the AV were the major contributor for the significant difference observed between TOTOX values of pollock oils distilled at the three tested evaporator temperatures.

### 4.2. Removal of Lipid Hydrolysis Products by SPD

The SPD process was very efficient at removing free fatty acids (FFA) from both hoki and pollock oils. This method removed high amounts (13.1 mg KOH/g) of FFA from the hoki oil. The SPD process removed even minor quantities (0.5 mg KOH/g) of FFA that were found in crude pollock oil to very low levels of 0.06–0.09 mg KOH/g recorded for distilled oils. The FFA of distilled pollock oils were comparable to the levels determined for distilled hoki oils (0.04–0.06 mg KOH/g). The distilled oils FFA concentrations were far beneath the suggested maximum levels of the GOED voluntary monograph (<3 mg KOH/g) [19]. Overall, the SPD system operated at the evaporator temperatures studied yielded virtually complete removal of FFA from crude oils, regardless of their initial FFA content.

It has previously been shown that FA ethyl esters added to fish oil assisted in the removal of persistent organic pollutants using SPD [26]. It was proposed that the addition of the FA ethyl esters led to the formation of a “working fluid” that enhanced the efficacy of the process [26]. The working fluid model can include any volatile compounds in the fish oils, including FA ethyl esters, FFA, cholesterol, mono-, di- and triacyl glycol, natural vitamins and antioxidants, and added antioxidants and carriers used in the commercial formulations (e.g., propylenglycol). The fluidity of this mixture will also depend on the internal condenser temperature. It is possible that the higher content of the FFA in the crude hoki oils assisted, via a similar mechanism, in the removal of the secondary oxidation products as determined by *p*-AV. This “working fluid” model may help explain the enhanced reduction in *p*-AV of distilled hoki oils as compared with the more gradual, and temperature-dependent, effect observed for the distilled pollock oil samples (Figure 2b). Even though data in this study does not support conclusive remarks about the applicability of the working fluid model to explain the research findings, it suggests further research in this particular topic should be conducted.

### 4.3. Effect of SPD on Oil Fatty Acid Composition

A major consideration for the use of SPD in fish oil processing is whether it affects the concentrations of EPA and DHA in the oil. Overall there were no appreciable changes to FA profiles in either of the oils for any of the temperatures tested. There were no differences in the concentrations of EPA and DHA in any oils post SPD treatment of the hoki oil. In the Pollock oil there was a small although significant increase in both DHA and EPA in the 210 °C fraction. The preservation of PUFA at all tested temperature conditions confirmed that SPD is an effective method to purify fish oils, which have been previous shown in other studies [26,28]. Further, as expected, SPD reduced the amount of unsaponifiable matter in the oil which has also been previously demonstrated [26]. The advantages of the SPD system include the short residence time of the oil in the evaporator combined with very low operating pressures and limited reaction time available for undesirable lipid degradation processes to take place, which are known to occur during distillation of oils when using traditional oil processing [5].

### 4.4. Uses of SPD in Marine Oil Processing

Liver oils, historically obtained from Atlantic cod, have been consumed in Scandinavian countries as far back as the middle ages and are an important source of *n*-3 LC-PUFA [21]. More recently pollock oils and oils from other gadoid species (which includes Hoki) and Hake have overtaken cod as the traditional source of fish oil [21]. This is the first reported use of SPD to purify hoki and pollock oils. SPD provides a rapid and gentle way to increase the quality and value of these oils. In a preliminary study (data not shown) it was determined that no significant difference (Tukey’s Honest Significant Difference Test; *p* < 0.05) existed in various quality parameters examined between pollock oil purified at 210 °C using roller speeds of 500 or 450 rpm. The 50 rpm decrease in the roller speed, from the maximum allowable setting of 500 rpm, was selected for purification of hoki oils because this speed poses less mechanical stress to the internal movable parts of the evaporator that operate the wiped film blades.

Preventing fish oil lipids from undergoing undesirable oxidative chemical changes during rendering and purification steps is a key element to obtaining a final product that has prolonged shelf life and adequate sensorial and nutritive properties. It also ensures consumer’s safety. It has been previously reported that SPD can strip oil of natural and added antioxidants [29,32]. A 50% reduction in tocopherol concentration was demonstrated by the application of a series of different SPD conditions in rapeseed oil [32]. In another study, reductions of up to 90% of the original concentrations of antioxidants were observed in vegetable oils subjected to short-path distillation [29]. It was suggested that through the application of antioxidants at different steps of oil processing, such as pre- and post-SPD, a reduction in the oxidation status of the oil can be achieved; however, the antioxidant systems used were not disclosed due to commercial sensitivities [29]. Liver oils such as hoki, pollock and cod are known to be a good source of fat soluble vitamins such as A, D and E. This may be advantageous if hoki or pollock oil are to be consumed on a daily basis to achieve intake of EPA and DHA at the levels recommended for certain disorders. For example, the American Heart Association’s (AHA) recommendation of consumption of 2–4 g of EPA+DHA per day would require 10 to 20 g of hoki and pollock oils. In these doses the amount of minor components of the oil such as lipid soluble vitamins may be approaching the upper limits (UL) of recommend dietary intake. Pollock oil lipid soluble vitamins have been reported with vitamin A (retinol) 103 g/g and vitamin E (measured as -tocopherol) 172 g/g [11]. Unrefined hoki oil has higher levels of vitamin A 1400–1900 g/g, vitamin E (measured as -tocopherol) 600–1100 g/g and ~100 g/g of vitamin D [39,40]. The recommended dietary intake (RDI) and UL, which is maximum daily intake unlikely to cause adverse health effects, for an adult male and female are different for each vitamin. The RDI from the nutrient reference values of Australia and New Zealand of vitamin A is 900 µg/day for men and 700 µg/day for women with a shared UL of 3,000 µg/day [41]. Two grams of hoki oil would reach the UL of vitamin A consumption, and similarly for vitamin D. However, for vitamin E the UL and RDI are substantially higher than the content in hoki oil (300 mg/g UL, 4 mg/g RDI). In this study we did not measure the effect of SPD on the vitamin content of the hoki and pollock oils, but it is expected that there could be a loss of lipid-soluble vitamins using this process. Previous work has seen vitamin loss in spratt (*Sprattus sprattus*) oil up to 82% for vitamin A, 64% vitamin D and 42% vitamin K [26]. The loss of lipid soluble in vitamins by SPD pollock and hoki oils would be important to establish for commercial use.

This work was carried out using a laboratory-scale bench top SPD. This equipment has several glass-on-glass connections which do not always give a good seal and require extensive leak checks and verification that stable pressure values have been achieved before distillation is carried out. All distilling pressures were <0.02 mbar and the vacuum varied slightly between replicate distillation runs, for instance the range of pressures recorded for nine pollock oil distillations was 0.018–0.011 mbar. This slight change in vacuum between runs due to the apparatus has been previously reported [29]; however, industrial scale stainless steel SPD equipment provides finer control of the vacuum between replicate distillations. Albeit not included in this study, determination of the concentration of antioxidants pre- and post-SPD purification of oils, together with other parameters such as sensory properties, polar lipids and oxidative stability trials, should be considered for future research and development endeavors on these oils.

## 5. Conclusions

The Alaskan fish oil industry is growing, with several companies refining marine oils and selling higher value products. However, there are still many companies that sell fish oil for non-edible purposes, such as for use in aquaculture. The New Zealand hoki industry does have the capability to make edible fish oils; however, the bulk of the oil is still sold as crude or unrefined due to processing costs. SPD provides a gentle and efficient way to improve the quality of these oils that could be readily added into the rendering processes in these countries. Moreover, SPD has the potential to provide major benefits for industry as it involves reduced and simplified processing for the purification of marine oils. Unfortunately, as is often the case for the new technologies, SPD involves high operating costs which have prevented the broad uptake of this technology by industry to date.

Although recognition of the importance of oil quality and sustainable processing is growing, potential cost saving and/or oil yield increases remain the prime parameters for the implementation of a new process. However, the potential for SPD to provide improved oil quality has been clearly demonstrated in this study.
